# Identification and quantification of putative active constituents of huangqin decoction for ulcerative colitis treatment through the integration of chemical profiling, network pharmacology, and bioinformatics

**DOI:** 10.3389/fchem.2026.1897628

**Published:** 2026-07-31

**Authors:** Seol Jang, Yu Jin Kim, Youn-Hwan Hwang

**Affiliations:** KM Convergence Research Division, Korea Institute of Oriental Medicine, Daejeon, Republic of Korea

**Keywords:** bioinformatics analysis, chemical constituents, Huangqin decoction, network pharmacology, UHPLC-Q-Orbitrap-MS, UPLC-TQ-MS/MS

## Abstract

**Introduction:**

Huangqin decoction (HQD), a traditional Chinese medicine prescription, is used to treat gastrointestinal diseases, including ulcerative colitis (UC). However, systematic research on the components of HQD remains insufficient. Therefore, we aimed to perform chemical profiling, network pharmacology, and bioinformatics analyses of HQD to identify candidate constituents potentially associated with UC and to establish a quantitative method for their determination in HQD.

**Methods:**

Qualitative chemical profiling was performed to identify 51 compounds in HQD, and their potential targets were predicted. UC-related target genes were identified by combining results from public and Gene Expression Omnibus (GEO) databases. Gene Ontology (GO) and Kyoto Encyclopedia of Genes and Genomes (KEGG) enrichment analyses were performed to investigate the biological processes and signaling pathways associated with UC. Moreover, protein–protein interaction (PPI) analysis was performed. Based on these results, 15 putative active constituents of HQD were selected and quantified. Molecular docking analysis was then performed to evaluate the binding interactions between these compounds and key target proteins.

**Results:**

A total of 947 HQD component-related, 1,868 UC-related, and 2,930 GEO database-related target genes were intersected to obtain 109 common target genes for HQD and UC. GO and KEGG enrichment analyses indicated that these targets were mainly associated with inflammatory and immune-related biological processes and signaling pathways. Among the identified targets, NOS2, AHR, MMP3, MMP9, and PRKCQ were highlighted as potential key targets. The analysis of three batches of HQD samples revealed that baicalin had the highest content. Molecular docking results indicated favorable predicted interactions between the putative active compounds and core target proteins, with several compound–target pairs exhibiting docking scores below −11.0 kcal/mol.

**Discussion:**

This study not only provides a comprehensive chemical profile of HQD but also presents a new approach for evaluating and managing quality based on putative active constituents. These findings may serve as a scientific basis for further pharmacological and clinical studies.

## 1 Introduction

Ulcerative colitis (UC) is a chronic inflammatory disorder characterized by inflammation of the colonic mucosal surface, with progression from mild to extensive inflammation and a clinical course of relapse and remission (Ordas et al., 2012; Ungaro et al., 2019). Patients with UC typically present with clinical symptoms of bloody stool and abdominal cramping and are at an increased risk of developing malignant tumors (Baumgart and Sandborn, 2007). UC significantly affects the quality of life of patients and imposes an economic burden (Kawalec, 2016). Although various chemical-based drugs and targeted therapies are available for clinical treatment, the global incidence of UC continues to rise, and these therapies are associated with potential side effects (Guo and Wang, 2023).

Traditional Chinese medicine (TCM) has been widely used in the treatment of UC because of its unique advantages, such as high efficacy and safety. TCMs with various targets and synergistic effects owing to their complex composition have been prescribed for UC treatment (Huang et al., 2021; Zhang et al., 2022). Huangqin decoction (HQD), a TCM prescription in Shang Han Lun, consists of four herbal medicines: *Scutellaria baicalensis* Georgi, *Glycyrrhiza uralensis* Fischer, *Paeonia lactiflora* Pallas, and *Zizyphus jujuba* Miller var. *Inermis* Rehder (Li et al., 2021). HQD has been used to treat gastrointestinal disorders for thousands of years and is effective in treating UC (Liu et al., 2022). A recent study investigated the clinical efficacy of HQD and western medicine combination therapy in treating pediatric patients with UC, reporting a low incidence of side effects and good therapeutic effects (Yinjuan et al., 2024). In addition, studies on the chemical constituents of HQD have investigated its pharmacological activity and quality (Li et al., 2016; Tang et al., 2024). TCM prescriptions, such as HQD, comprise various herbal medicines containing abundant chemical components; therefore, identifying their components is very important for clinical application and to ensure safety and consistency (Tang et al., 2024). However, most studies have focused on verifying the efficacy of HQD in UC using animal experiments (Li et al., 2022; Li et al., 2019; Li et al., 2023), and systematic research on their components is insufficient.

Network pharmacology, which is based on a systems biology approach, is a core methodology in modern TCM research that systematically elucidates the pharmacological actions and safety of TCMs, enabling the transition from experience-based to evidence-based medicine (Zhang et al., 2019). Moreover, it effectively investigates TCM mechanisms in disease treatment and is widely used to explore disease and drug mechanisms and active ingredients (Shen et al., 2023; Zheng et al., 2022). Previous studies have reported the therapeutic effects of some TCM prescriptions, including HQD, on UC using network pharmacology (Duan et al., 2023; Huang et al., 2021; Li et al., 2021; Xu et al., 2021); however, most have explored the potential mechanism of treatment or identified the mechanism using animal models. Moreover, most studies aimed at identifying active compounds for the treatment of UC have relied on database-based predictions, whereas studies based on the actual chemical constituents present in the extracts are insufficient. Therefore, a more accurate and practical component analysis of the TCM formulations is required.

The analysis of TCM preparations is not only difficult from an analytical perspective because of its complexity, but also requires safety, making liquid chromatography-mass spectrometry-based analytical methods increasingly important (Ganzera and Sturm, 2018). Ultra-high-performance liquid chromatography/quadrupole Orbitrap mass spectrometry (UHPLC-Q-Orbitrap-MS) is an effective, rapid, and excellent analytical method for identifying chemical components in complex herbal formulations owing to its high sensitivity and specificity (Zhang et al., 2023). In addition, ultra-performance liquid chromatography coupled with triple-quadrupole tandem mass spectrometry (UPLC-TQ-MS/MS) can simultaneously detect various components within a short analysis time, providing systematic information on compounds in complex matrices (Gao et al., 2021). Therefore, in this study, we conducted a qualitative analysis of HQD extracts using UHPLC-Q-Orbitrap-MS, followed by network pharmacology analysis based on the identified compounds to select putative active constituents against UC treatment, which were then quantitatively analyzed using UPLC-TQ-MS/MS.

Bioinformatic analysis is used to elucidate and explore the mechanisms of various diseases and may help discover new biomarkers for improving the diagnosis and prognosis of UC (Cheng et al., 2020). Additionally, collecting gene expression data using microarray technology and identifying differentially expressed genes (DEGs) through bioinformatics analysis can provide important insights into the pathogenesis of UC and contribute to the derivation of stable and reliable results (Shi et al., 2020; Zhu et al., 2019). Therefore, in this study, we combined the results from multiple disease databases with those obtained from DEGs derived from the Gene Expression Omnibus (GEO) database to identify key targets for UC.

Moreover, it is important to develop a more comprehensive quality control (QC) method to ensure the quality and safety of herbal medicines prescribed for disease treatment (Wang et al., 2021). However, systematic and comprehensive studies on the chemical components of HQD are lacking. Therefore, in this study, we identified UC disease targets and selected putative active constituents by combining network pharmacology and bioinformatics analysis based on the chemical profiling of HQD. In addition, we quantified the putative active constituents using an optimized UPLC-TQ-MS/MS analysis method, through which we established an integrated system that enables the quality evaluation and management of HQD. Furthermore, molecular docking analysis was performed to evaluate the potential interactions between the putative active constituents and UC-related target proteins and to provide computational support for the predicted compound–target relationships. In the future, this study is expected to provide a reliable QC method for HQD, verify its therapeutic effect on UC, and help elucidate its therapeutic mechanism.

## 2 Materials and methods

### 2.1 Chemicals and sample preparation

#### 2.1.1 Chemicals and reagents

All standards used for the qualitative and quantitative analyses were purchased from ChemFaces (Wuhan, China) and TargetMol (Wellesley Hill, MA, United States). Warfarin (internal standard (IS)) was purchased from Sigma-Aldrich (St. Louis, MO, United States), and the purity of all standards was >98%. Methanol, acetonitrile, and formic acid were all MS grade and were purchased from Thermo Fisher Scientific (Loughborough, United Kingdom).

#### 2.1.2 Preparation of HQD extract

*Scutellaria baicalensis* (66.5 g), *Z. jujuba* (66.5 g), *P. lactiflora* (50.0 g), and *G. uralensis* (50.0 g) were weighed and mixed. Thereafter, 10 times the total weight of water was added, refluxed at 100 °C for 3 h, and filtered. The filtrate was concentrated under reduced pressure and freeze-dried to obtain the HQD extract (yield: 38.79%). The four herbal medicines in HQD, *S. baicalensis*, *Z. jujuba*, *P. lactiflora*, and *G. uralensis,* were purchased from Kwangmyungdang Pharmaceutical (Ulsan, Republic of Korea), and voucher specimens for each were deposited in the KM Convergence Research Division, Korea Institute of Oriental Medicine (Specimen No. FHM-K-02, FHM-K-09–11).

#### 2.1.3 Sample and standard solution

Standard stock solutions of 15 compounds were accurately weighed and individually dissolved in methanol. Each stock solution was mixed and diluted with methanol to obtain standard solutions. A mixed standard solution consisting of 15 compounds was diluted with methanol to create a working solution with an appropriate concentration for use as a calibration curve and QC sample. QC samples were prepared at low, medium, and high concentrations for the recovery efficiency and method validation. HQD-1, HQD-2, and HQD-3 represent three independently prepared sample solutions obtained from the same HQD extract and were analyzed to evaluate analytical reproducibility. The sample stock solution was prepared by dissolving 20 mg of HQD extract powder in 1 mL of methanol, followed by sonication for 30 min and centrifugation at 12,500 rpm for 15 min. Prior to UPLC-TQ-MS/MS analysis, the stock solution was diluted 20,000-fold with methanol to obtain a final analytical concentration of 0.001 mg/mL. The same dilution factor was applied to all analytes, and all analyte responses were confirmed to fall within the validated calibration ranges. Warfarin was selected as the IS because it exhibited stable chromatographic retention, reproducible multiple reaction monitoring (MRM) responses, efficient ionization in both positive and negative ionization modes, and no detectable interference with the target analytes under the optimized UPLC-TQ-MS/MS conditions. The internal standard was added at a constant concentration (5 ng/mL) to the standard and sample solutions used for analysis.

### 2.2 Chemical profiling and quantitative analysis

#### 2.2.1 UHPLC-Q-Orbitrap-MS

The constituent compounds of HQD were identified using a Dionex UltiMate 3000 system equipped with a Thermo Q-Exactive mass spectrometer (Thermo Fisher Scientific, Waltham, MA, United States). All analytes were separated on an Acquity BEH C18 column (100 × 2.1 mm, 1.7 µm, Waters, Milford, MA, United States), and the column temperature was maintained at 40 °C. The mobile phase consisted of A (0.1% formic acid in water) and B (acetonitrile), with a gradient elution of 3% B for 0–1 min, 3%–15% B for 1–2 min, 15%–50% B for 2–13 min, 50%–100% B for 13–20 min, 100% B for 20–23 min, 100%–3% B for 23–23.5 min, and 3% B for 23.5–27.5 min. The mobile phase was set at a flow rate of 0.25 mL/min, and the injection volume of all analytes was 3.0 µL. Mass data were acquired in both positive and negative ion modes using electrospray ionization in the full MS-ddMS2 scan mode. The full scan range was 100–1,500 m*/z,* and the scan resolution was set to 70,000 for MS and 17,500 for MS/MS. The normalized collision energy required to obtain fragment ions was set to 25 eV. Data acquisition and processing were performed using Xcalibur v.3.0 and Tracefinder v.3.2 software (Thermo Fisher Scientific, Waltham, MA, United States).

#### 2.2.2 UPLC-TQ-MS/MS

An Agilent 1290 Infinity II system coupled to an Agilent 6495C triple-quadrupole mass spectrometer with a jet-stream electrospray ionization source (Agilent Technologies, Santa Clara, CA, United States) was used for quantitative analysis. The separation conditions for chromatographic analysis were the same as those used for the qualitative analysis. Fifteen compounds were detected by MRM in both positive and negative ion modes. The MRM parameters for each compound were optimized, and the selected values are summarized in Table 1. Agilent MassHunter software (v.10.1) was used for the acquisition and processing of all experimental data.

**TABLE 1:** Optimized MRM parameters for analytes and IS in UPLC-TQ-MS/MS analysis.

No.	Compound name	Rt (min)	Ion mode	MRM transitions (*m/z*)	Collision energy (V)	Cell accelerator (V)	Fragmentor (V)
				Q1 → Q3			
1	Magnoflorine	4.63	Positive	342.2 → 58.2	30	4	166
2	Albiflorin	4.94	Positive	481.2 → 105.0	22	4	166
3	Paeoniflorin	5.23	Negative	525.2 → 449.1	10	4	166
4	Liquiritin apioside	5.67	Negative	549.2 → 255.1	34	4	166
5	Scutellarin	5.78	Positive	463.0 → 287.0	22	4	166
6	Acteoside	5.80	Negative	623.2 → 161.0	40	4	166
7	Liquiritin	5.81	Negative	417.2 → 255.0	18	4	166
8	Baicalin	7.79	Positive	447.1 → 271.0	22	4	166
9	Ononin	7.89	Positive	431.1 → 269.1	18	4	166
10	Wogonoside	9.32	Positive	461.1 → 285.1	22	4	166
11	Benzoylpaeoniflorin	9.77	Negative	629.1 → 553.2	10	4	166
12	Baicalein	10.89	Positive	271.0 → 123.0	34	4	166
13	Glycyrrhizic acid	12.01	Negative	821.4 → 351.0	40	4	166
14	Wogonin	13.02	Positive	285.1 → 270.0	26	4	166
15	Oroxylin A	13.58	Positive	285.0 → 270.0	26	4	166
IS1	Warfarin	14.00	Negative	307.0 → 250.0	22	4	166
IS2	Warfarin	14.00	Positive	309.0 → 163.0	14	4	166

MRM, multiple reaction monitoring; UPLC-TQ-MS/MS, ultra-performance liquid chromatography coupled with triple-quadrupole tandem mass spectrometry; IS, internal standards.

#### 2.2.3 Method validation

The quantitative method was validated for the selected analytes in terms of linearity, lower limit of quantification (LLOQ), accuracy, precision, and recovery. The validation strategy followed a fit-for-purpose approach for comparative quantification of major constituents in HQD samples. System suitability was confirmed by five consecutive injections of the mixed standard solution before sample analysis to ensure stable chromatographic and mass spectrometric performance. Calibration curves were constructed using multiple concentration levels spanning the expected analytical ranges of the target compounds. The mixed stock solution containing 15 analytes was serially diluted stepwise with methanol to prepare calibration working solutions at eight concentration levels, each of which was analyzed five times. Calibration curves were constructed using the peak area ratio of each analyte to the IS over the validated concentration ranges. Linearity was evaluated using weighted (1/x^2^) linear regression analysis to generate the calibration equations. The lowest measured concentration that allowed accuracy and precision was selected as the lower limit of quantification. To evaluate the precision and accuracy of the analytical method, QC samples were analyzed on one and three consecutive days. The relative standard deviation (RSD%) and accuracy (%) of the QC samples were used to evaluate the intra- and inter-day precision and accuracy of the method. The acceptance criteria were set as RSD < 15% for intra- and inter-day precision, with RSD < 20% allowed at the LLOQ, and accuracy within ±15% of the nominal concentration (Shah et al., 2000). Precision and accuracy at the LLOQ were evaluated together with the QC samples. Recovery tests were performed by spiking HQD samples with 15 analytes at low, medium, and high concentrations, and the added and detected amounts of each analyte were compared to calculate recovery. Blank samples were analyzed before and during sample analysis to monitor carryover, and no significant carryover was observed.

### 2.3 Network pharmacology analysis

#### 2.3.1 Prediction of target genes for chemical components

For the chemical components of the HQD identified in the qualitative analysis, SMILES structures were confirmed in PubChem (https://PubChem.ncbi.nlm.nih.gov/), and potential targets corresponding to each component were predicted using the Swiss Target Prediction (http://www.swisstargetprediction.ch/, accessed on 25 November 2025) database. Compounds that were not available in SwissTargetPrediction were further searched in the STITCH database (http://stitch.embl.de/, accessed on 25 November 2025). The species was restricted to *Homo sapiens*, and all predicted targets, including those with a prediction probability of zero, were retained for subsequent analysis. Target names were standardized to official gene symbols, and duplicate targets were removed prior to network construction. The complete compound–target association table is provided in Supplementary Table S1.

#### 2.3.2 Acquisition of UC-related target genes

UC-related target genes were searched on four databases using the keyword “ulcerative colitis”: OMIM database (https://omim.org/, accessed on 6 August 2025), GeneCards database (https://www.genecards.org/, accessed on 6 August 2025), DisGeNET database (https://www.disgenet.org/, accessed on 6 August 2025), and TTD database (https://idrblab.net/ttd/, accessed on 6 August 2025). In GeneCards, only genes with a relevance score ≥ 5 were retained. The UC-related target genes from the databases were merged, and duplicates were removed. Data on the remaining target genes were collected.

#### 2.3.3 Data acquisition and analysis of DEGs

Gene expression profiles associated with UC were obtained from the GEO, a Public Functional Genomics Data Repository. Two GEO datasets (GSE92415 and GSE107499) were selected, and gene expression profiles in tissues of patients with UC and healthy controls were obtained. The GSE92415 dataset (GPL13158 platform) contained 162 and 21 colonic mucosal biopsy samples from patients with UC and healthy controls, respectively. The GSE107499 dataset (GPL15207 platform) contained 75 inflammatory colonic tissues from patients with active UC and 44 normal colonic tissues. DEGs between UC and normal control samples were identified using the GEO2R online tool. Adjusted *P*-values were calculated using the Benjamini–Hochberg false discovery rate correction implemented in GEO2R. Statistical significance was defined as |Log_2_FC| > 0.5 and an adjusted *P*-value < 0.05, and the DEGs were visualized using volcano plots. Probe identifiers were mapped to gene symbols according to the corresponding GEO platform annotation files. When multiple probes corresponded to the same gene symbol, duplicate entries were removed prior to subsequent analyses. DEGs common to both datasets were identified using the online tool VENNY 2.1 based on gene-symbol intersections, and only genes showing consistent directions of differential expression in both datasets were retained as UC-related target genes.

#### 2.3.4 Gene Ontology (GO) and kyoto encyclopedia of genes and genomes (KEGG) pathway analysis

The target genes of the compounds and diseases, and the results of the GEO analysis, were combined, and duplicated target genes were derived using Venn diagrams. The selected target genes were subjected to GO and KEGG enrichment analysis using the online tool Enrichr (https://amp.pharm.mssm.edu/Enrichr/), and the top 20 items were selected based on the *P*-value for each item. GO and KEGG pathway enrichment analysis was performed using Enrichr’s GO Biological Process 2025 and KEGG 2021 Human libraries (accessed on 17 March 2026).

#### 2.3.5 Protein–protein interaction (PPI) network analysis

Based on the GO and KEGG enrichment results, 12 UC-related terms associated with inflammation, immune regulation, and signaling pathways were selected. A total of 61 genes associated with these selected terms were used to construct the PPI network using the Search Tool for the Retrieval of Interacting Genes (STRING) database (http://string-db.org/). *Homo sapiens* was selected as the species, and the minimum required interaction score was set to 0.400 (medium confidence). Active interaction sources included textmining, experiments, databases, co-expression, neighborhood, gene fusion, and co-occurrence. No additional interactors beyond the query proteins were included, and disconnected nodes were retained in the network. Subsequently, the interaction network between the selected genes and HQD components was constructed and visualized using Cytoscape 3.10.2, and putative active constituents involved in the treatment of UC were selected.

### 2.4 Molecular docking

We evaluated binding interactions between putative active compounds and target proteins through molecular docking analysis. Target proteins associated with the 15 quantitatively analyzed compounds were collected, and only proteins with available three-dimensional structures in the Protein Data Bank (PDB) were selected for molecular docking analysis. The 3D structures of the target proteins were obtained from the RCSB Protein Data Bank (http://www.rcsb.org/), and the 3D structures of the putative active compounds were obtained from the PubChem Compound Database (https://pubchem.ncbi.nlm.nih.gov/). Detailed information on the protein structures used for docking is provided in Supplementary Table S2. Protein structures were prepared by removing water molecules and adding hydrogen, whereas ligand structures underwent an energy minimization process prior to docking. Molecular docking was performed using AutoDock Vina implemented in PyRx (version 0.8). Blind docking was performed using grid boxes covering the entire protein structures, and the binding energies generated by the interactions were calculated. The docking results were evaluated using the lowest binding-energy pose generated by AutoDock Vina. The docking results of the putative active compounds and target proteins were visualized as heatmaps, and for structures with some low binding energies, the docking results were visualized using PyMOL 3.1.8 software.

## 3 Results

### 3.1 Identification of chemical components and target genes in HQD

**FIGURE 1:**
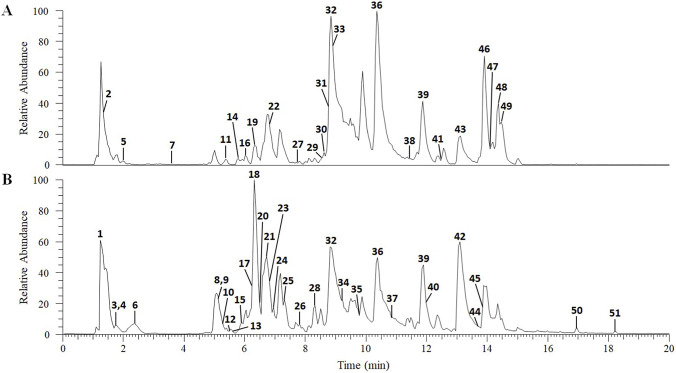
Base peak ion chromatograms of the HQD extract obtained in positive **(A)** and negative **(B)** ion modes using UHPLC-Q-Orbitrap-MS. The identification numbers of the various phytochemicals are listed in Table 2. HQD, Huangqin decoction; UHPLC-Q-Orbitrap-MS, ultra-high-performance liquid chromatography/quadrupole Orbitrap mass spectrometry.

The chemical components of the HQD extract were identified using UHPLC-Q-Orbitrap-MS, and the base peak chromatograms analyzed in positive and negative ion modes are shown in Figure 1. A total of 51 compounds were identified as HQD components, and the information related to each compound is summarized in Table 2. Among them, 35 compounds were unambiguously identified by comparison with the corresponding reference compounds, and 16 compounds were tentatively identified by referring to the literature. The identified compounds included 31 flavonoids, four monoterpenoids, four alkaloids, four triterpenoids, two phenolic compounds, two phenylpropanoids, one saponin, and three other compounds. In addition, based on previously reported studies, the identified compounds were assigned to the constituent herbal medicines of HQD (Li et al., 2021; Tang et al., 2024). Among the 51 identified compounds, 15 were associated with *S. baicalensis*, eight with *Z. jujuba*, eight with *P. lactiflora*, and 20 with *G. uralensis*. A total of 947 compound-related targets were identified from 51 compounds, and detailed data on compound-target associations are provided in Supplementary Table S1.

**TABLE 2:** Identification of chemical constituents in HQD using UHPLC-Q-Orbitrap-MS.

No.	Rt (min)	Identification	Formula	Adduct	Expected (*m/z*)	Measured (*m/z*)	Error (ppm)	MS^2^ fragment (*m/z*)	Source
1	1.24	Maltose^*^	C_12_H_22_O_11_	[M − H]^−^	341.1089	341.1098	2.4676	89	ZJ
2	1.35	Proline^*^	C_5_H_9_NO_2_	[M + H]^+^	116.0706	116.0712	4.7987	70	GU
3	1.73	Citric acid	C_6_H_8_O_7_	[M − H]^−^	191.0197	191.0195	−1.4399	111	ZJ
4	1.77	8-Debenzoylpaeoniflorin	C_16_H_24_O_10_	[M + HCO_2_]^−^	421.1352	421.1362	2.5656	345, 165	PL
5	1.98	Adenosine^*^	C_10_H_13_N_5_O_4_	[M + H]^+^	268.1040	268.1046	2.0654	136	ZJ, GU, PL
6	2.37	Gallic acid^*^	C_7_H_6_O_5_	[M − H]^−^	169.0142	169.0137	−3.3303	169, 125	ZJ, PL
7	3.61	Phenylalanine^*^	C_9_H_11_NO_2_	[M + H]^+^	166.0863	166.0866	2.1006	120	GU
8	5.12	Oxypaeoniflorin	C_23_H_28_O_12_	[M − H]^−^	495.1508	495.1518	1.9467	137	PL
9	5.17	Catechin^*^	C_15_H_14_O_6_	[M − H]^−^	289.0718	289.0725	2.6993	289, 245	ZJ, PL
10	5.30	Glucoliquiritin apioside	C_32_H_40_O_18_	[M − H]^−^	711.2142	711.2158	2.2923	549, 255	GU
11	5.37	Licoagroside B	C_18_H_24_O_12_	[M + H]^+^	433.1340	433.1350	2.1454	127	GU
12	5.49	Vicenin-2^*^	C_27_H_30_O_15_	[M − H]^−^	593.1512	593.1530	3.0770	593, 473, 353	GU
13	5.63	Epicatechin^*^	C_15_H_14_O_6_	[M − H]^−^	289.0718	289.0728	3.5438	289, 245	ZJ
14	5.79	Magnoflorine^*^	C_20_H_24_NO_4_	[M]^+^	342.1700	342.1707	1.9706	342, 297	ZJ
15	5.90	Schaftoside^*^	C_26_H_28_O_14_	[M − H]^−^	563.1406	563.1422	2.7007	563, 353	GU, PL, SB
16	6.02	Albiflorin^*^	C_23_H_28_O_11_	[M + H]^+^	481.1704	481.1714	2.0944	105	PL
17	6.22	Ganhuangemin	C_15_H_12_O_7_	[M − H]^−^	303.0510	303.0519	2.8044	177, 125	SB
18	6.31	Paeoniflorin^*^	C_23_H_28_O_11_	[M + HCO_2_]^−^	525.1614	525.1627	2.5892	121	PL
19	6.34	Chavicol	C_9_H_10_O	[M + H]^+^	135.0804	135.0807	1.9524	105, 79	ZJ
20	6.49	Rutin^*^	C_27_H_30_O_16_	[M − H]^−^	609.1461	609.1475	2.3180	300, 223, 131	ZJ
21	6.72	Liquiritin apioside^*^	C_26_H_30_O_13_	[M − H]^−^	549.1614	549.1624	1.9203	549, 255	GU
22	6.84	Scutellarin^*^	C_21_H_18_O_12_	[M + H]^+^	463.0871	463.0877	1.2460	287	SB
23	6.86	Liquiritin^*^	C_21_H_22_O_9_	[M − H]^−^	417.1191	417.1200	2.1220	255, 135	GU
24	6.95	Acteoside^*^	C_29_H_36_O_15_	[M − H]^−^	623.1981	623.1995	1.9250	623, 461, 161	SB
25	7.31	Isoacteoside^*^	C_29_H_36_O_15_	[M − H]^−^	623.1981	623.1992	1.7310	623, 461, 161	SB
26	7.81	Sophoraflavone B	C_21_H_2_0O_9_	[M − H]^−^	415.1035	415.1044	2.1925	295	GU
27	8.07	Iridin^*^	C_24_H_26_O_13_	[M + H]^+^	523.1446	523.1459	2.3971	361	SB
28	8.32	Isoliquiritin apioside	C_26_H_30_O_13_	[M − H]^−^	549.1614	549.1630	2.9206	255	GU
29	8.53	Scutellarein^*^	C_15_H_10_O_6_	[M + H]^+^	287.0550	287.0554	1.3217	287	SB
30	8.62	Isoliquiritin^*^	C_21_H_22_O_9_	[M + H]^+^	419.1337	419.1341	1.1088	257	GU
31	8.75	Viscidulin III	C_17_H_14_O_8_	[M + H]^+^	347.0761	347.0765	1.1470	347	SB
32	8.84	Baicalin^*^	C_21_H_18_O_11_	[M + H]^+^	447.0922	447.0927	1.1684	271	SB
33	8.94	Ononin^*^	C_22_H_22_O_9_	[M + H]^+^	431.1337	431.1343	1.5735	269	GU
34	9.23	Liquiritigenin^*^	C_15_H_12_O_4_	[M − H]^−^	255.0663	255.0668	2.2298	255, 135, 119	GU
35	9.78	Licorice glycoside E	C_35_H_35_NO_14_	[M − H]^−^	692.1985	692.2003	2.6544	549, 255, 160	GU
36	10.36	Wogonoside^*^	C_22_H_2_0O_11_	[M + H]^+^	461.1078	461.1084	1.2773	285	SB
37	10.84	Benzoylpaeoniflorin^*^	C_30_H_32_O_12_	[M + HCO_2_]^−^	629.1876	629.1891	2.3950	121	PL
38	11.46	Glabrolide	C_30_H_44_O_4_	[M + H]^+^	469.3312	469.3321	1.8160	469, 175	GU
39	11.89	Baicalein^*^	C_15_H_10_O_5_	[M − H]^−^	269.0456	269.0464	3.1100	269	SB
40	12.03	Isoliquiritigenin^*^	C_15_H_12_O_4_	[M − H]^−^	255.0663	255.0668	2.2298	255, 135, 119	GU
41	12.47	Formononetin^*^	C_16_H_12_O_4_	[M + H]^+^	269.0808	269.0813	1.7052	269	GU
42	13.10	Glycyrrhizic acid^*^	C_42_H_62_O_16_	[M − H]^−^	821.3965	821.3988	2.7925	351, 193, 113	GU
43	13.12	Zizyberenalic acid	C_30_H_44_O_3_	[M + H]^+^	453.3363	453.3370	1.5797	453	ZJ
44	13.70	Amentoflavone	C_30_H_18_O_10_	[M − H]^−^	537.0827	537.0842	2.6951	537, 391, 245	GU
45	13.84	Licorice saponin B2	C_42_H_64_O_15_	[M − H]^−^	807.4172	807.4192	2.4144	351	GU
46	13.91	Wogonin^*^	C_16_H_12_O_5_	[M + H]^+^	285.0758	285.0761	1.2658	285, 270	SB
47	14.09	Chrysin^*^	C_15_H_10_O_4_	[M + H]^+^	255.0652	255.0655	1.1790	255	SB
48	14.37	Skullcapflavone II	C_19_H_18_O_8_	[M + H]^+^	375.1074	375.1079	1.3347	375	SB
49	14.46	Oroxylin A^*^	C_16_H_12_O_5_	[M + H]^+^	285.0758	285.0761	1.1587	285, 270	SB
50	16.97	Glabridin^*^	C_20_H_2_0O_4_	[M − H]^−^	323.1289	323.1298	2.9146	323, 201, 135	GU
51	18.20	Hederagenin^*^	C_30_H_48_O_4_	[M − H]^−^	471.3480	471.3491	2.2914	471	PL

* Compared with the reference standard. ZJ: *Zizyphus jujuba* Miller var. *Inermis* Rehder; GU: *glycyrrhiza uralensis* fischer; PL: *paeonia lactiflora* pallas; SB: *scutellaria baicalensis* georgi.

HQD, huangqin decoction; UHPLC-Q-Orbitrap-MS, ultra-high-performance liquid chromatography/quadrupole Orbitrap mass spectrometry.

### 3.2 Network pharmacology analysis

#### 3.2.1 Prediction of UC-related target genes

**FIGURE 2:**
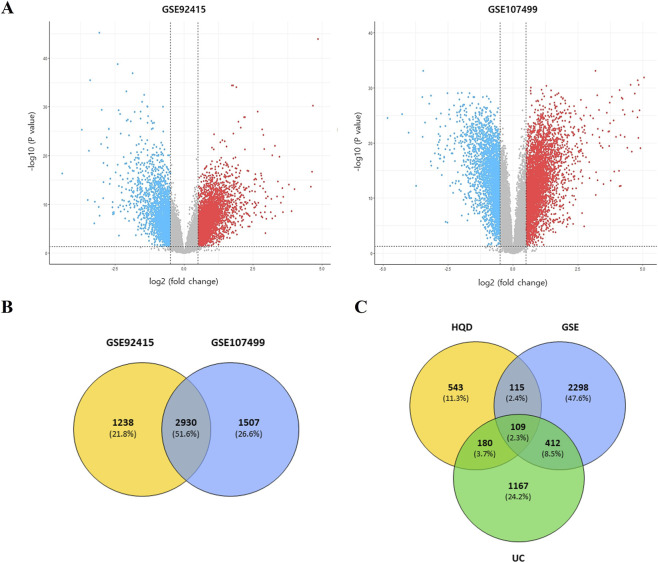
Prediction of UC-related target genes. Volcano plot of differentially expressed genes (DEGs) in the two datasets (GSE92415 and GSE107499). Red dots represent genes with upregulated expression, blue dots represent genes with downregulated expression, and gray dots represent genes with no significant difference **(A)**. Venn diagrams of overlapping DEGs between two datasets **(B)** and common target genes of HQD component and UC **(C)**. UC, ulcerative colitis; HQD, Huangqin decoction.

UC-related target genes were searched for in four databases: OMIM with 186 genes, GeneCards with 841 genes, DisGeNET with 1,458 genes, and TTD with 46 genes. By merging the results from each database and removing duplicates, 1,868 UC-related target genes were identified. DEGs were identified between patients with UC and healthy controls in two datasets from the GEO database. From the GSE92415 and GSE107499 datasets, 4,172 and 4,441 genes were extracted, respectively, and the DEGs analysis results for each dataset were plotted as a volcano map (Figure 2A). GSE92415 identified 1,670 upregulated and 2,502 downregulated genes (Supplementary Table S3), whereas GSE107499 identified 1,914 upregulated and 2,527 downregulated genes (Supplementary Table S4). A total of 2,930 common DEGs showing consistent directions of differential expression across the two GEO datasets were identified (Figure 2B). Finally, 947 HQD component-related targets, 1,868 UC-related targets, and 2,930 GEO database-related targets were intersected to obtain 109 common HQD and UC target genes (Figure 2C; Supplementary Table S5).

#### 3.2.2 GO and KEGG pathway analysis

**FIGURE 3:**
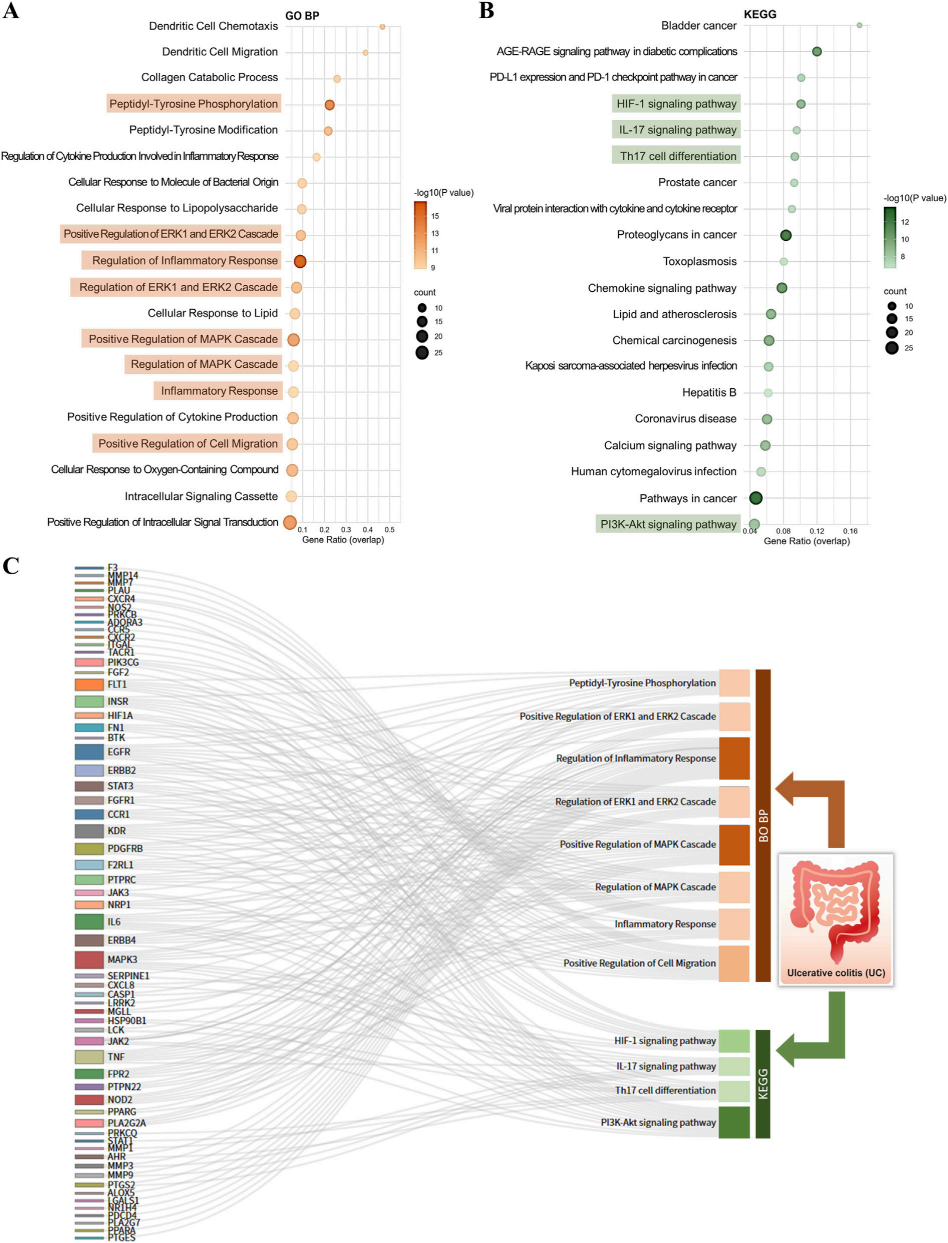
Gene Ontology (GO) **(A)** and Kyoto Encyclopedia of Genes and Genomes (KEGG) **(B)** enrichment analysis of target genes. Bubble plot of the top 20 enriched GO biological process (BP) terms for the common target genes **(A)** and the top 20 enriched KEGG pathways **(B)**. Sankey diagram illustrating the relationships between target genes, selected GO BP terms, and KEGG pathways **(C)**.

GO enrichment analyses were performed on 109 duplicate target genes, and the top 20 BP terms were extracted based on statistical significance. Among them, we selected eight representative terms related to inflammation and signaling pathways, including inflammatory response, peptidyl-tyrosine phosphorylation, positive regulation of cell migration, positive regulation of the ERK1/ERK2 cascade, positive regulation of the MAPK cascade, regulation of the ERK1/ERK2 cascade, regulation of inflammatory response, and regulation of the MAPK cascade (Figure 3A). KEGG pathway analysis demonstrated that target genes appear significantly abundant in various biological pathways. Among these, pathways previously reported to be associated with inflammation, immune regulation, and the pathogenesis of ulcerative colitis were selected for focused analysis (Liu et al., 2021; Wu et al., 2022). These include HIF-1, IL-17, and PI3K-Akt signaling pathways, as well as Th17 cell differentiation. (Figure 3B). The complete GO and KEGG enrichment results generated by Enrichr are provided in Supplementary Tables S6, S7. A target–pathway network was constructed to more clearly demonstrate the relationship between target genes and the GO and KEGG pathways (Figure 3C). As shown in Figure 3C, several target genes are simultaneously associated with various pathways, including HIF-1, IL-17, and PI3K–Akt signaling pathways, as well as major biological processes such as inflammatory responses and MAPK/ERK signaling.

#### 3.2.3 PPI network analysis and putative active constituents prediction

**FIGURE 4:**
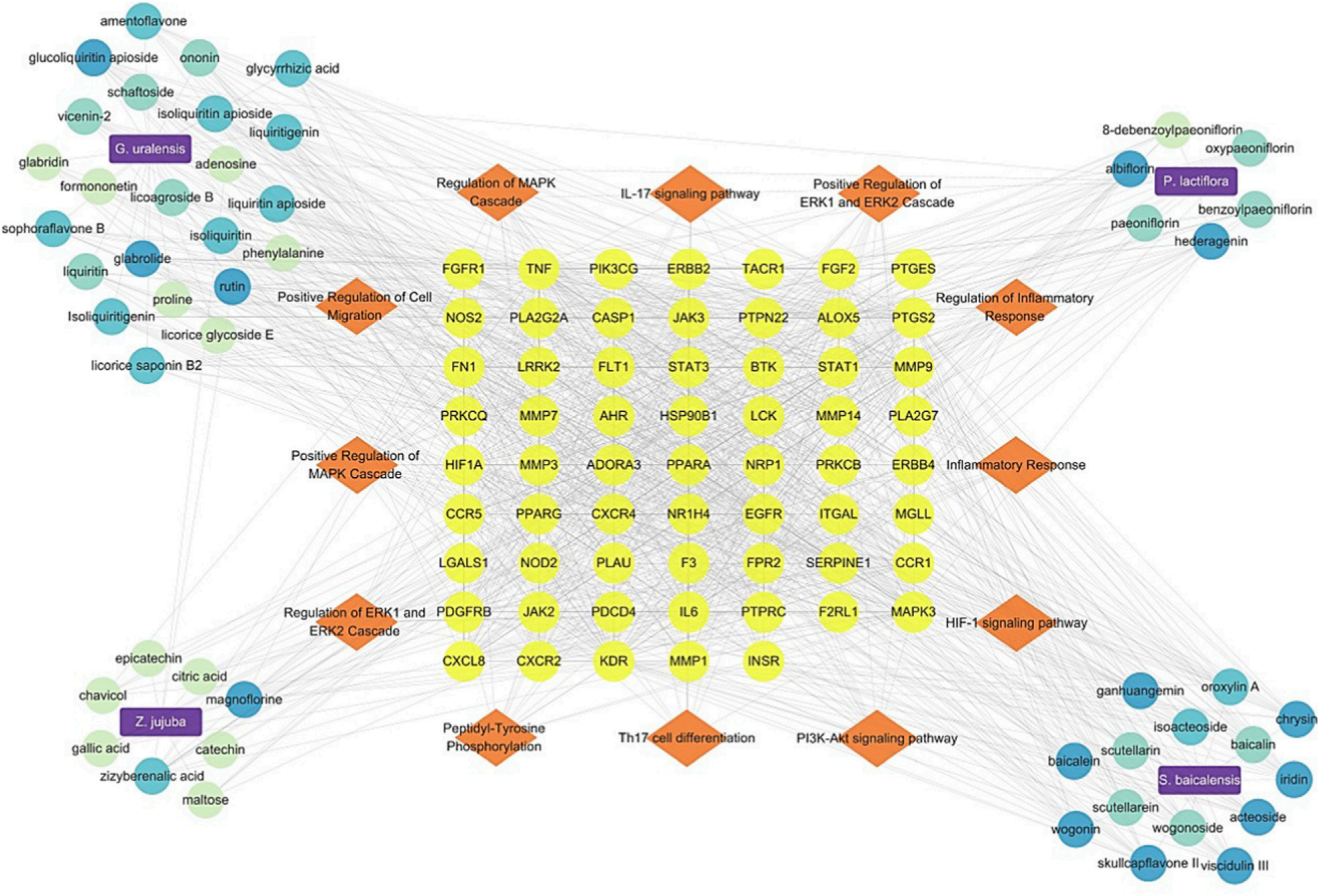
Compound–target–pathway network of HQD. Purple nodes represent the herbal medicines in HQD, blue nodes represent chemical compounds, yellow nodes represent target genes, and orange diamond-shaped nodes represent GO and KEGG pathways. Edges indicate compound–target interactions or associations between target genes and GO and KEGG pathways. HQD, Huangqin decoction.

To perform a detailed analysis of overlapping target genes between HQD and UC, we first selected 12 UC-related terms from the top 20 terms identified in the GO and KEGG pathway analysis. We analyzed the PPI network of 61 genes (Supplementary Table S8) associated with the selected terms using the STRING database. Subsequently, we constructed the network relationships between the selected genes and HQD components using Cytoscape software. As shown in Figure 4, the network consists of 128 nodes and 1,309 edges. Purple square nodes represent the four herbal medicines constituting HQD, blue circle nodes represent the 51 compounds identified in HQD, yellow circle nodes represent 61 related target genes, and orange diamond nodes represent the 12 GO and KEGG pathways. Edges indicate interactions between compounds and target genes, or associations between target genes and pathways. The results showed that there is a complex correlation between compounds and targets, and the darker the color of the circle-shaped node representing the compound, the more interactions there are between the compounds and the targets. For example, albiflorin, baicalein, and magnoflorin were associated with 13 targets, acteoside and wogonin with 12 targets, and oroxylin A with 11 targets. These results suggested that specific compounds are associated with multiple targets and are likely to be the main putative active constituents of HQD involved in the treatment of UC. Based on the network analysis results, 15 compounds (magnoflorine, albiflorin, paeoniflorin, liquiritin apioside, scutellarin, acteoside, liquiritin, baicalin, ononin, wogonoside, benzoylpaeoniflorin, baicalein, glycyrrhizic acid, wogonin, and oroxylin A) were selected as putative active constituents of the HQD among the compounds linked to more than seven targets based on the qualitative analysis results.

### 3.3 Quantitative analysis of putative active constituents of HQD

#### 3.3.1 Quantitative analysis

**FIGURE 5:**
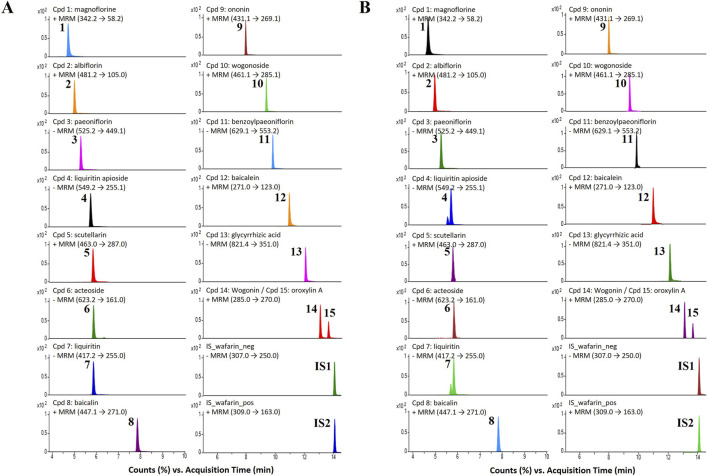
Representative MRM chromatograms of the reference standard solution **(A)** and HQD extract **(B)** used for the quantitative determination of 15 compounds by UPLC-TQ-MS/MS. UPLC-TQ-MS/MS, ultra-performance liquid chromatography coupled with triple-quadrupole tandem mass spectrometry; MRM, multiple reaction monitoring; HQD, Huangqin decoction.

To analyze the putative active constituents of HQD, 15 compounds were simultaneously quantified using UPLC-TQ-MS/MS. All compounds were detected within approximately 15 min. The total chromatographic run time was approximately 27.5 min, including the column washing and re-equilibration steps described in the gradient program. The detected compounds were distributed in each herbal medicine constituting the HQD as follows: scutellarin, acteoside, baicalin, wogonoside, baicalein, wogonin, and oroxylin A from *S. baicalensis*; magnoflorine from *Z. jujuba*; albiflorin, paeoniflorin, and benzoylpaeoniflorin from *P. lactiflora*; and liquiritin apioside, liquiritin, ononin, and glycyrrhizic acid from *G. uralensis*. The compounds identified in this study have also been reported as major constituents of HQD in previous chemical profiling studies (Li et al., 2016; Li et al., 2019). Dynamic MRM was performed for accurate and sensitive quantitative analysis. The precursor ions, product ions, and collision energy were optimized in the MRM mode by considering the characteristic parameters of each compound, and the detailed analysis conditions are summarized in Table 1. All compounds were analyzed individually in positive and negative ion modes according to detection sensitivity, with magnoflorine, albiflorin, scutellarin, baicalin, ononin, wogonoside, baicalein, wogonin, and oroxylin A showing higher detection intensities in the positive ion mode than in the negative ion mode. The remaining compounds were selected in negative ion mode, which showed higher detection intensities than those in positive ion mode. Representative MRM chromatograms of the reference standard solution and HQD extract used for the quantitative analysis of the 15 compounds are shown in Figure 5.

#### 3.3.2 Method validation

To validate the UPLC-TQ-MS/MS method for analyte quantification, linearity, precision, accuracy, and recovery were investigated. A calibration curve was generated using the peak area (Y) and concentration (X) of each analyte and the IS. The linear range was selected based on the concentration of each analyte in the sample, and good linearity was observed, with a correlation coefficient (R^2^) ranging from 0.9990 to 0.9994. The lower limit of quantification of 15 analytes was in the range of 0.02–1.56 ng/mL, and the detailed results are presented in Table 3. Precision was evaluated using the RSD; the RSD of the intra-day precision was in the range of 0.27%–6.28%, and the RSD of the inter-day precision was in the range of 0.25%–8.09%. Additionally, the accuracy evaluation results were in the range of 90.14%–109.79% for intra-day and 91.17%–104.54% for inter-day. The average recovery rate of 15 analytes was in the range of 91.61%–107.81%, and the RSD was in the range of 0.36%–3.87%. As presented in Table 4, the developed quantitative analysis method showed good precision, accuracy, and recovery, indicating its suitability for accurate quantification of 15 putative active compounds.

**TABLE 3:** Linear regression and LLOQ values of 15 analytes in HQD for method validation.

No.	Compound name	Calibration curves	Linear range (ng/mL)	R^2^	LLOQ (ng/mL)
1	Magnoflorine	y = 0.431028x − 0.002095	0.05–12.50	0.9990	0.05
2	Albiflorin	y = 0.011666x − 0.000133	0.05–12.50	0.9994	0.05
3	Paeoniflorin	y = 0.058974x − 0.004921	0.39–100.00	0.9993	0.39
4	Liquiritin apioside	y = 0.094094x − 0.001184	0.20–50.00	0.9993	0.20
5	Scutellarin	y = 0.009589x − 0.000028	0.02–6.25	0.9990	0.02
6	Acteoside	y = 0.039244x − 0.000634	0.05–12.50	0.9992	0.05
7	Liquiritin	y = 0.161083x − 0.000885	0.10–25.00	0.9992	0.10
8	Baicalin	y = 0.019967x + 0.000524	1.56–400.00	0.9994	1.56
9	Ononin	y = 0.116863x − 0.001513	0.05–12.50	0.9992	0.05
10	Wogonoside	y = 0.015843x − 0.006822	1.56–400.00	0.9992	1.56
11	Benzoylpaeoniflorin	y = 0.051781x − 0.000331	0.02–6.25	0.9992	0.02
12	Baicalein	y = 0.014169x + 0.000632	0.10–25.00	0.9993	0.10
13	Glycyrrhizic acid	y = 0.026484x − 0.001693	0.39–100.00	0.9994	0.39
14	Wogonin	y = 0.118617x − 0.003641	0.10–25.00	0.9991	0.10
15	Oroxylin A	y = 0.127588x − 0.001964	0.05–12.50	0.9991	0.05

LLOQ, lower limit of quantification; HQD, huangqin decoction.

**TABLE 4:** Precision, accuracy, and recovery of 15 analytes in HQD for method validation.

No.	Compound name	Concentration (ng/mL)	Precision (RSD, %)		Accuracy (%)		Recovery (%)	
			Intra-day	Inter-day	Intra-day	Inter-day	Mean	RSD
1	Magnoflorine	0.52	0.90	3.78	97.10	94.87	92.18	1.11
		2.08	0.94	0.52	93.60	93.32	92.07	0.84
		8.33	0.45	2.06	95.59	94.22	99.20	2.42
2	Albiflorin	0.52	1.65	5.46	109.78	103.39	102.31	3.47
		2.08	1.50	5.86	107.83	104.54	106.86	0.63
		8.33	0.34	3.50	101.30	99.10	106.46	1.11
3	Paeoniflorin	4.17	1.47	5.91	106.06	99.30	93.08	0.86
		16.67	1.35	7.80	104.80	101.83	100.26	0.37
		66.67	0.39	3.60	99.95	96.76	99.42	1.27
4	Liquiritin apioside	2.08	0.88	3.74	104.01	99.93	93.91	1.31
		8.33	0.46	6.57	108.47	101.13	95.17	0.63
		33.33	0.95	3.31	99.91	97.08	103.88	1.78
5	Scutellarin	0.26	6.28	5.67	95.23	100.60	94.99	0.92
		1.04	2.74	1.82	90.14	91.64	91.61	2.03
		4.17	0.47	2.28	96.79	94.53	106.08	2.44
6	Acteoside	0.52	1.92	3.53	100.68	97.41	92.22	1.63
		2.08	1.17	3.85	99.33	95.15	92.16	3.89
		8.33	1.98	1.30	96.05	95.29	94.21	2.78
7	Liquiritin	1.04	1.02	4.17	106.37	102.17	92.18	0.65
		4.17	1.96	7.51	109.79	101.58	99.15	1.11
		16.67	1.19	4.05	98.88	96.81	104.28	1.30
8	Baicalin	16.67	0.27	6.67	97.92	101.04	93.92	0.76
		66.67	0.84	0.25	91.40	91.17	91.88	2.39
		266.67	1.06	2.06	92.70	94.72	105.51	0.68
9	Ononin	0.52	1.24	1.40	98.10	99.05	97.51	0.79
		2.08	1.10	4.81	100.78	99.30	101.63	1.24
		8.33	0.72	0.99	99.80	98.67	104.97	0.39
10	Wogonoside	16.67	0.34	2.94	99.58	97.07	93.89	1.69
		66.67	1.78	2.92	92.76	93.88	91.97	1.54
		266.67	0.98	3.95	92.12	96.04	107.36	0.98
11	Benzoylpaeoniflorin	0.26	3.70	1.69	101.74	99.82	96.91	1.72
		1.04	1.76	7.85	109.14	102.01	101.18	0.97
		4.17	2.30	4.19	98.73	95.68	100.24	0.47
12	Baicalein	1.04	1.20	8.09	93.45	102.30	106.37	3.18
		4.17	1.65	1.72	91.13	92.98	95.33	2.98
		16.67	0.53	5.35	99.44	97.75	105.43	1.47
13	Glycyrrhizic acid	4.17	1.35	3.65	98.43	94.62	96.21	1.61
		16.67	0.83	1.21	100.79	99.43	98.10	0.61
		66.67	1.30	3.12	94.34	94.80	97.73	0.36
14	Wogonin	1.04	1.68	4.97	92.19	97.16	92.67	1.16
		4.17	0.72	2.66	92.18	93.80	101.51	2.12
		16.67	1.10	3.28	93.22	96.86	107.81	1.71
15	Oroxylin A	0.52	1.42	2.87	93.20	96.25	92.83	1.50
		2.08	0.47	0.97	92.30	92.29	102.42	0.75
		8.33	1.15	3.48	91.54	94.81	105.88	1.54

HQD, huangqin decoction; RSD, relative standard deviation.

#### 3.3.3 Sample analysis

The contents of 15 putative active constituents were analyzed in three batches of HQD samples using a validated quantitative analytical method. The detailed results are presented in Table 5. Baicalin and wogonoside showed the highest content with a range of 61.87–64.45 mg/g, followed by liquiritin apioside and glycyrrhizic acid with a range of 8.09–8.62 mg/g.

**TABLE 5:** Quantitative analysis of 15 analytes in three batches of HQD.

No.	Compound	Content (Mean ± SD, mg/g)		
		HQD-1 (n = 6)^*^	HQD-2 (n = 6)	HQD-3 (n = 6)
1	Magnoflorine	2.399 ± 0.050	2.533 ± 0.028	2.487 ± 0.046
2	Albiflorin	1.449 ± 0.033	1.449 ± 0.031	1.506 ± 0.045
3	Paeoniflorin	14.359 ± 0.087	14.485 ± 0.084	14.528 ± 0.069
4	Liquiritin apioside	8.553 ± 0.066	8.621 ± 0.075	8.537 ± 0.044
5	Scutellarin	0.923 ± 0.009	0.966 ± 0.024	0.967 ± 0.021
6	Acteoside	1.431 ± 0.038	1.817 ± 0.074	1.821 ± 0.083
7	Liquiritin	2.295 ± 0.042	2.311 ± 0.056	2.230 ± 0.084
8	Baicalin	63.140 ± 0.823	63.937 ± 0.858	64.451 ± 0.923
9	Ononin	1.367 ± 0.010	1.345 ± 0.010	1.362 ± 0.030
10	Wogonoside	62.065 ± 0.706	61.873 ± 0.682	63.162 ± 0.809
11	Benzoylpaeoniflorin	0.419 ± 0.005	0.428 ± 0.005	0.417 ± 0.005
12	Baicalein	3.681 ± 0.064	3.763 ± 0.098	3.896 ± 0.069
13	Glycyrrhizic acid	8.086 ± 0.054	8.199 ± 0.067	8.238 ± 0.051
14	Wogonin	2.476 ± 0.040	2.625 ± 0.034	2.561 ± 0.042
15	Oroxylin A	1.101 ± 0.014	1.197 ± 0.029	1.166 ± 0.042

* Replicate injections. HQD, huangqin decoction; SD, standard deviation.

### 3.4 Molecular docking analysis between putative active constituents and targets

**FIGURE 6:**
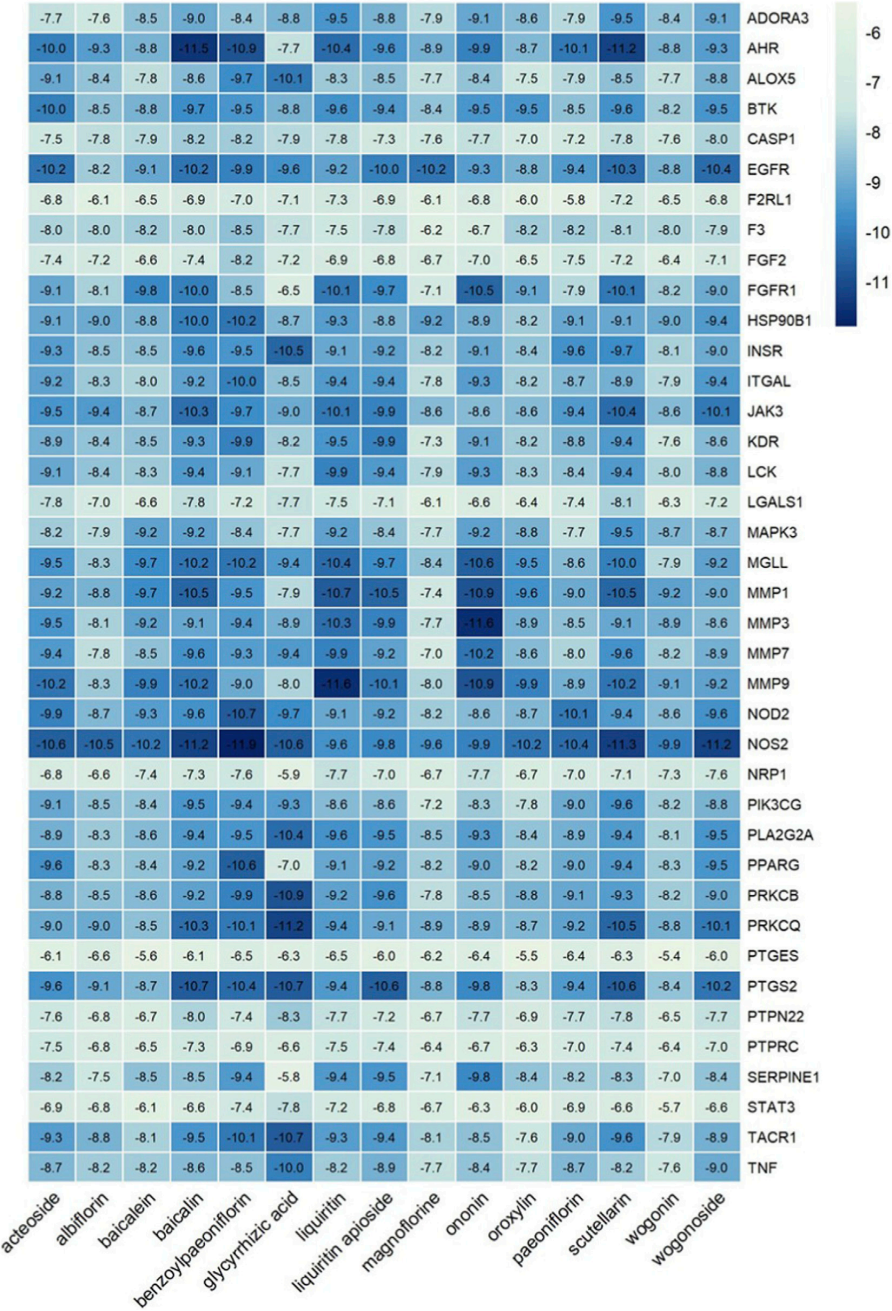
Heat map of molecular docking results between putative active compounds and target proteins. Rows represent target proteins, and columns represent putative active compounds. Darker colors indicate stronger binding affinity.

**FIGURE 7:**
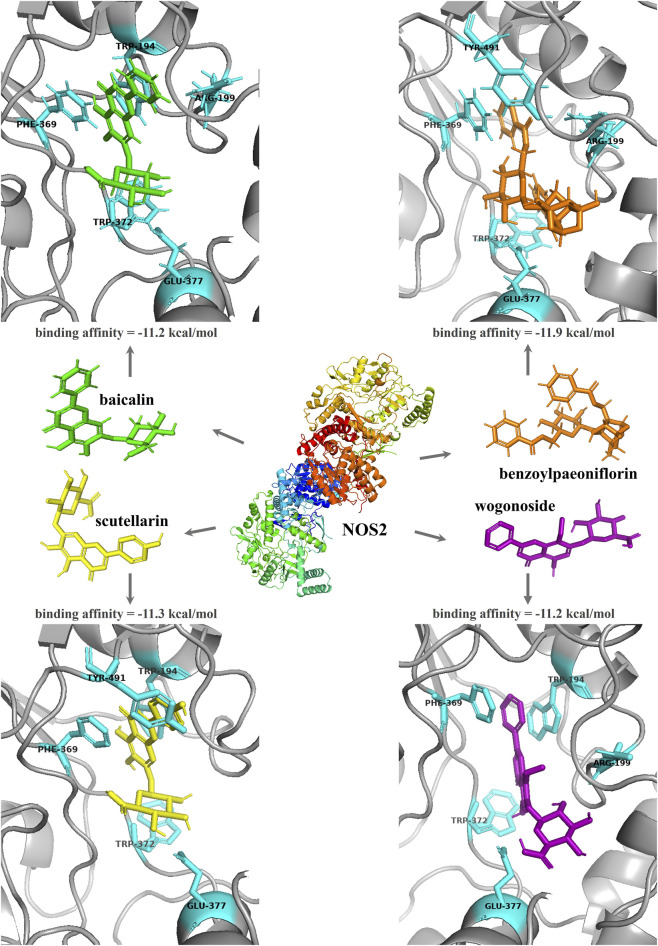
Molecular docking interactions between representative putative active compounds and NOS2. The binding modes of baicalin, benzoylpaeoniflorin, scutellarin, and wogonoside are shown.

**FIGURE 8:**
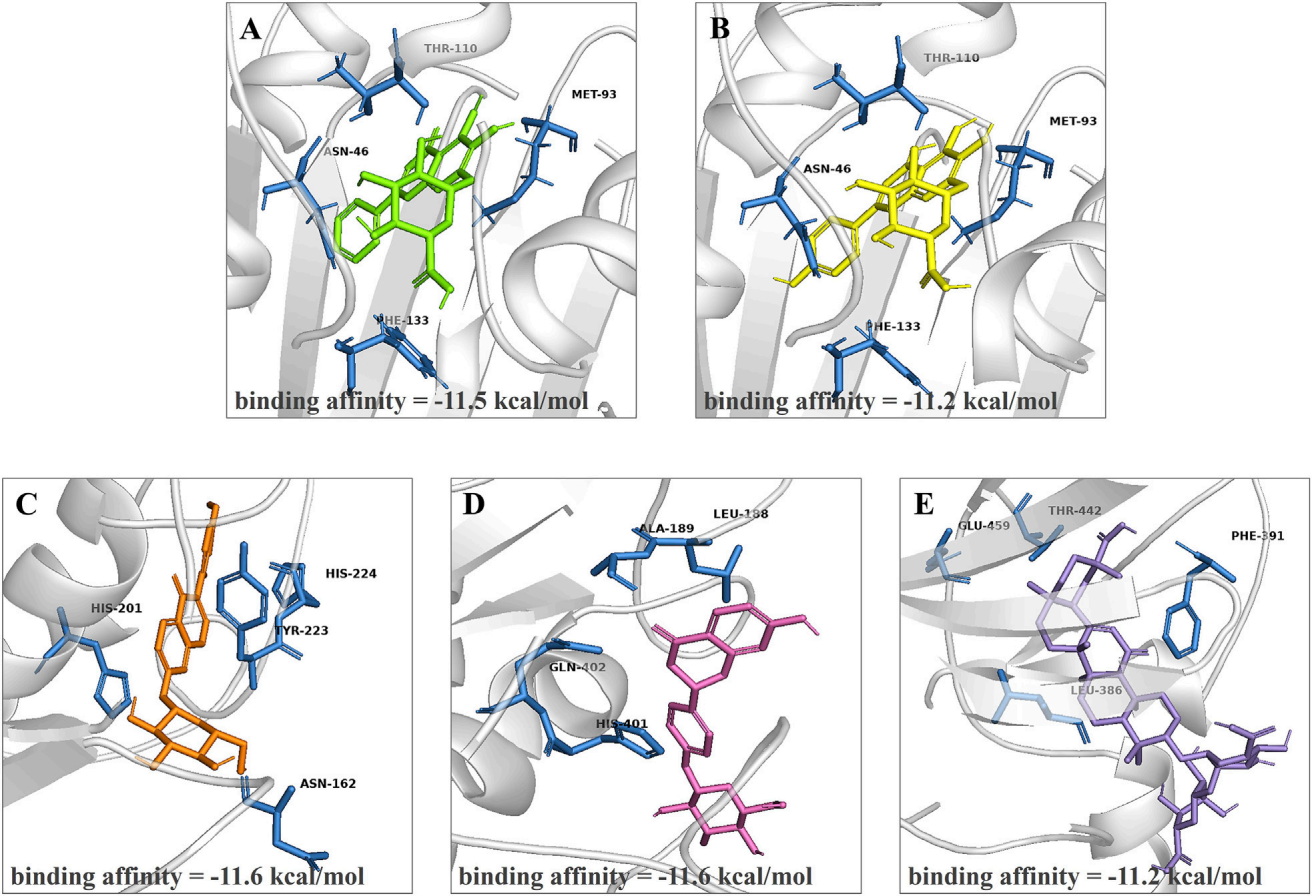
Molecular docking results of putative active compounds and target proteins: baicalin-AHR **(A)**, scutellarin-AHR **(B)**, ononin-MMP3 **(C)**, liquiritin-MMP9 **(D)**, glycyrrhizic acid-PRKCQ **(E)**.

Molecular docking was performed as a complementary computational approach to assess the binding potential of the putative active constituents of HQD toward UC-related target proteins identified through network pharmacology analysis. Based on the results, molecular docking was conducted between 15 candidate compounds and 39 target proteins, and docking scores were calculated for each compound-protein pair. The calculated docking scores were used to estimate the potential interactions between ligands and target proteins, with lower docking scores indicating more favorable predicted binding under the selected docking conditions. As shown in Figure 6, all compound–protein pairs yielded docking scores below −5.0 kcal/mol, suggesting potentially favorable predicted binding affinities between the selected compounds and target proteins. Among the analyzed target proteins, NOS2 showed relatively favorable docking scores with most of the selected candidate compounds. In particular, baicalin, benzoylpaeoniflorin, scutellarin, and wogonoside exhibited docking scores below −11.0 kcal/mol against NOS2. The interaction between the ligand and the receptor was primarily stabilized by several key amino acid residues, including phenylalanine (PHE369), tryptophan (TRP372), and glutamic acid (GLU377), which were commonly observed in all four compounds (Figure 7; Supplementary Figure S1). In addition, AHR–baicalin (Figure 8A), AHR–scutellarin (Figure 8B), MMP3–ononin (Figure 8C), MMP9–liquiritin (Figure 8D), and PRKCQ–glycyrrhizic acid (Figure 8E) also exhibited relatively favorable docking scores below −11.0 kcal/mol, supporting the potential interactions predicted by the network pharmacology analysis (Supplementary Figure S2).

## 4 Discussion

Although many studies have demonstrated that HQD is effective in treating UC, most have been limited to *in vitro* and animal experiments, and systematic and comprehensive studies on the components of HQD remain lacking (Wang et al., 2017). In this study, we utilized network pharmacology to select putative active constituents of HQD for UC treatment and established a quantitative analysis method, thereby increasing our understanding of the effective ingredients of HQD. First, 51 chemical compounds present in HQD were identified using UHPLC-Q-Orbitrap-MS. Comprehensive identification of chemical components provides a solid foundation for performing network pharmacology, and based on this, it enables the exploration of putative active constituents through integrated analysis with network pharmacology (Luo et al., 2019; Zhu et al., 2024). In addition, we identified DEGs in patients with UC using the GEO database to analyze biological information and derived genes related to UC. These results have enabled the discovery of potential targets for the diagnosis and treatment of UC, as biomarkers for an accurate diagnosis of UC are still lacking (Pan et al., 2023; Shi et al., 2020). Finally, 947 targets corresponding to compounds identified in HQD, 1,868 UC-related targets, and 2,930 GEO database-related targets were intersected to obtain 109 common target genes associated with for HQD and UC.

Based on these 109 common target genes, GO BP enrichment analysis revealed that these genes are primarily associated with inflammatory responses and MAPK/ERK signaling-related processes. These findings suggest that the therapeutic effects of HQD on UC may involve the regulation of intestinal inflammatory responses. Indeed, inflammatory responses have been reported to play a significant role in the pathogenesis of UC (Strober and Fuss, 2011). The MAPK/ERK signaling pathway plays a crucial role in regulating inflammatory responses by generating inflammatory cytokines and modulating immune cell activation (Saggini, 2024). Previous studies have reported that inhibition of the MAPK/ERK signaling pathway can significantly alleviate colonic inflammation and mucosal damage in UC mouse models (Gao et al., 2019). Additionally, the MAPK signaling pathway is known to be an important regulator of the inflammatory process in inflammatory diseases such as UC (Zhao et al., 2026). Therefore, the regulation of the MAPK/ERK signaling pathway may be an important mechanism supporting the therapeutic effects of HQD on UC. KEGG pathway analysis revealed that common targets are involved in immune and inflammation-related pathways, particularly the IL-17 signaling pathway and Th17 cell differentiation. Previous studies have shown that Th17 cell numbers and IL-17 levels are significantly elevated in patients with UC and positively correlate with disease severity (Wedebye Schmidt et al., 2013). Furthermore, it has been reported that intestinal inflammation in UC mouse models can be alleviated through the regulation of Th17 cell differentiation and the IL-17 signaling pathway (Li et al., 2025). Moreover, the PI3K–Akt and HIF-1 signaling pathways have been associated with common targets and reported to alleviate symptoms of intestinal diseases through the regulation of inflammatory processes (Di Mattia et al., 2024; Niu and Zhang, 2025). These results suggest that HQD can exert therapeutic effects on UC by regulating immune responses and major intracellular signaling pathways.

In this study, we selected terms related to UC from the GO and KEGG pathway enrichment analysis results and constructed a PPI network for target genes extracted from these terms. We then performed a network analysis of the compounds identified in HQD and the target genes of UC, and finally selected 15 putative active constituents of HQD by considering their association with multiple targets. The bioactive effects of the selected compounds on UC are supported by several previous studies. Magnoflorine, albiflorin, scutellarin, baicalin, and ononin effectively alleviate UC by reducing the production of inflammatory cytokines and regulating signal transduction pathways related to inflammation in dextran sulfate sodium-induced UC mice (Li et al., 2024; Wang et al., 2023; Wen et al., 2024; Yu et al., 2023; Zhang et al., 2017). In addition, paeoniflorin, acteoside, liquiritin, wogonoside, and baicalein have protective effects on the intestinal epithelial barrier function, suggesting that they can ameliorate colitis (Huang et al., 2020; Li et al., 2022; Liu et al., 2022; Ma et al., 2023; Zhang et al., 2022). Liquiritin apioside ameliorates gastrointestinal diseases by regulating intestinal metabolites, whereas glycyrrhizic acid ameliorates colonic inflammation and inhibits the development of colitis-related colorectal cancer (Chen et al., 2025; Xia et al., 2023). In addition, benzoylpaeoniflorin and wogonin are useful in the treatment of inflammatory diseases by inhibiting the expression of inflammatory mediators (Kim et al., 2022; Zhou et al., 2022). Finally, HQD quality was assessed by measuring the content of 15 putative active constituents, and a UPLC-TQ-MS/MS method was established for their quantitative analysis. The putative active ingredient contents in the three batches of HQD were measured, and the results showed that the contents of baicalin and wogonoside were the highest. In a previously reported clinical study on patients with UC, baicalin was shown to regulate immune balance and alleviate inflammatory responses caused by UC by promoting the proliferation of CD4^+^CD29^+^ cells (Yu et al., 2014).

The molecular docking results provided computational support for the potential interactions between the candidate bioactive constituents and target proteins predicted by the network pharmacology analysis. All selected compounds yielded docking scores below −5.0 kcal/mol with their corresponding targets, suggesting potentially favorable interactions under the selected docking conditions. These findings support the potential compound–target associations identified through network analysis and indicate that these interactions may contribute to the therapeutic effects of HQD. Among the identified targets, NOS2 showed relatively favorable docking scores with several compounds. In particular, baicalin and wogonoside, which were present at relatively high levels in the quantitative analysis of HQD, exhibited docking scores below −11.0 kcal/mol against NOS2. NOS2 is an important inflammatory mediator involved in the pathophysiology of UC. Previous studies have shown that NOS2 expression is significantly increased in UC models and that NOS2 inhibition can alleviate the progression of UC by regulating inflammatory signaling pathways (Wu et al., 2024; Zhang et al., 2022). Therefore, the results of this study suggest that major compounds of HQD, such as baicalin and wogonoside, may play an important role in the treatment of UC by regulating NOS2 activity. In addition to NOS2, several other target proteins exhibited strong binding affinities with specific putative active compounds. For example, AHR showed strong interactions with baicalin and scutellarin, and the regulation of AHR is associated with anti-inflammatory and immunomodulatory effects (Stockinger et al., 2024). MMP3 and MMP9 showed strong binding affinities with ononin and liquiritin, respectively, and MMPs have been reported to play important roles in mucosal degradation and inflammation in inflammatory bowel disease (Maronek et al., 2021). Additionally, PRKCQ exhibited strong binding affinity with glycyrrhizic acid, and PKC*θ* is known to play an important role in regulating T cell-mediated colitis and intestinal inflammation (Nagahama et al., 2008). Collectively, these findings suggest that the candidate bioactive constituents of HQD may exhibit therapeutic effects on UC by targeting key regulators of inflammation and immune responses, including NOS2, AHR, MMPs, and PRKCQ. However, as molecular docking is a predictive method based on static structure, it is insufficient to assess these interactions and biological efficacy. Furthermore, the predicted targets were derived from public databases, and the bioavailability of the identified compounds was not evaluated in this study. Therefore, additional experimental studies, including *in vitro* and *in vivo* studies, are required to confirm the pharmacological effects and mechanisms of action of HQD.

These results demonstrate that the proposed method can be extended beyond simple quality assessments based on the quantitative analysis of compounds within a prescription. In this study, we integrated network pharmacology and molecular docking to evaluate the quality of HQD based on putative active constituents and to explore the potential compound–target relationships associated with UC. In particular, the favorable docking scores further supported the predicted compound–target relationships. These results suggest that the identified putative active constituents may contribute to the therapeutic effects of HQD on UC and that the established analytical method is reliable for both quality assessment and the characterization of putative active constituents. However, this study is limited to the prediction of putative active constituents and potential targets. Therefore, further experimental verification using cell and animal models is necessary to elucidate the mechanism of action of HQD on UC and confirm the involvement of the proposed pathways and targets.

## 5 Conclusion

In this study, we identified 51 compounds in HQD using UHPLC-Q-Orbitrap-MS profiling. Based on the identified composition, we combined network pharmacology and bioinformatic analyses to identify 15 putative active constituents closely related to UC-related target genes. We established a quantitative analysis method for these putative active constituents and measured their contents in HQD extracts. In addition, molecular docking analysis predicted favorable binding interactions between these putative active compounds and key target proteins, providing computational support for the predicted compound–target interactions. These results provide a quantitative analytical approach for the characterization of HQD constituents and offer preliminary insights into the chemical basis of HQD associated with its potential therapeutic effects against UC. Further studies are needed to elucidate the mechanism of action of HQD in UC treatment and experimentally validate its therapeutic effects.

## Data Availability

The raw data supporting the conclusions of this article will be made available by the authors, without undue reservation.
