# Supplementary material for: Identification and quantification of putative active constituents of huangqin decoction for ulcerative colitis treatment through the integration of chemical profiling, network pharmacology, and bioinformatics
Source: Front Chem. 2026 Jul 31;14:1897628. doi: 10.3389/fchem.2026.1897628 (PMC13473006; doi:10.3389/fchem.2026.1897628)
Supplement: Supplementary file 1 [file Image1.pdf]

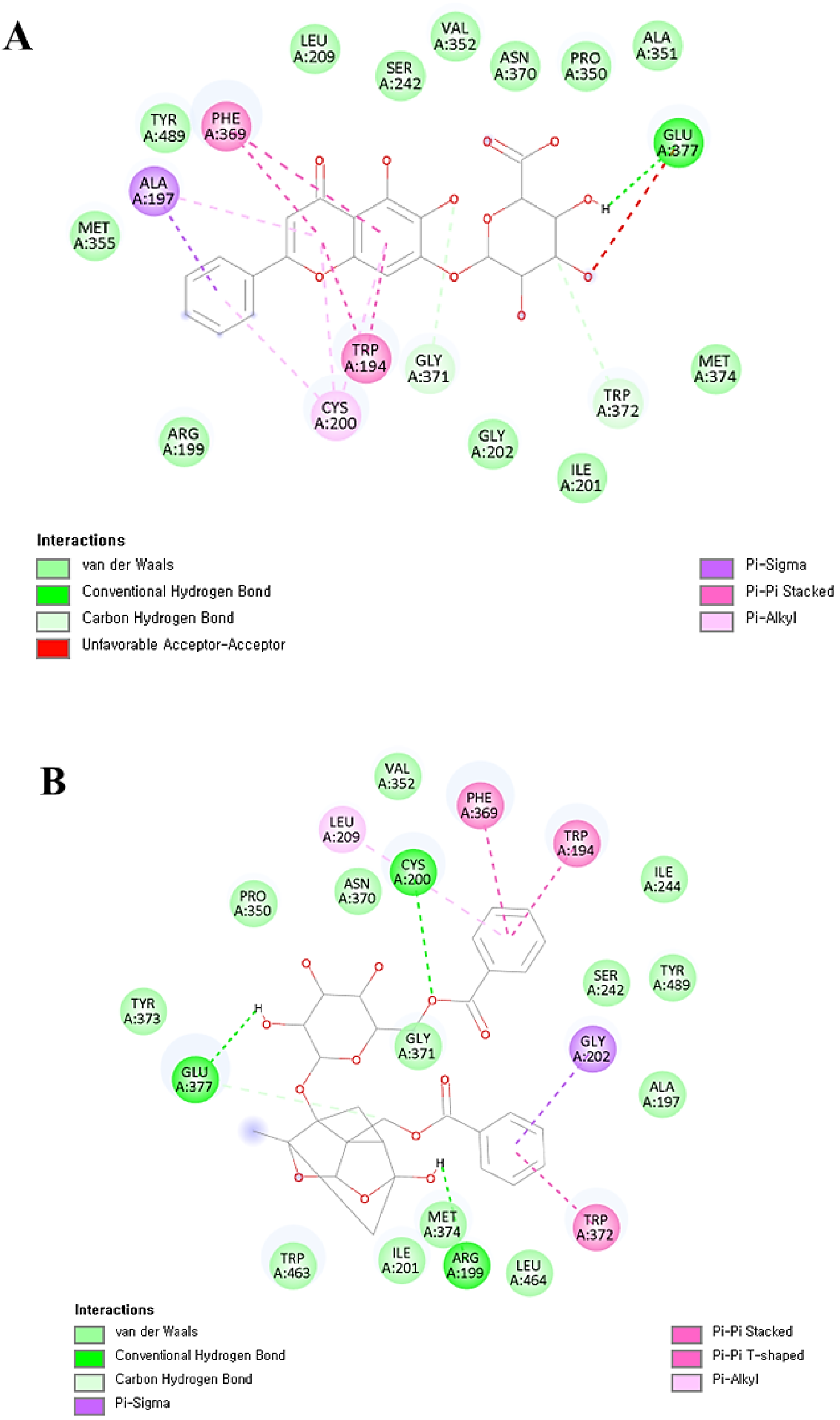
A
LEU A:209
VAL A:352
SER A:242
ASN A:370
PRO A:350
ALA A:351
TYR A:489
PHE A:369
GLU A:377
ALA A:197
MET A:355
TRP A:194
GLY A:371
MET A:374
CYS A:200
TRP A:372
ARG A:199
GLY A:202
ILE A:201
Interactions
van der Waals
Conventional Hydrogen Bond
Carbon Hydrogen Bond
Unfavorable Acceptor-Acceptor
Pi-Sigma
Pi-Pi Stacked
Pi-Alkyl
B
VAL A:352
PHE A:369
LEU A:209
TRP A:194
CYS A:200
ASN A:370
ILE A:244
PRO A:350
SER A:242
TYR A:489
TYR A:373
GLY A:371
GLY A:202
GLU A:377
ALA A:197
MET A:374
TRP A:372
TRP A:463
ILE A:201
ARG A:199
LEU A:464
Interactions
van der Waals
Conventional Hydrogen Bond
Carbon Hydrogen Bond
Pi-Sigma
Pi-Pi Stacked
Pi-Pi T-shaped
Pi-Alkyl

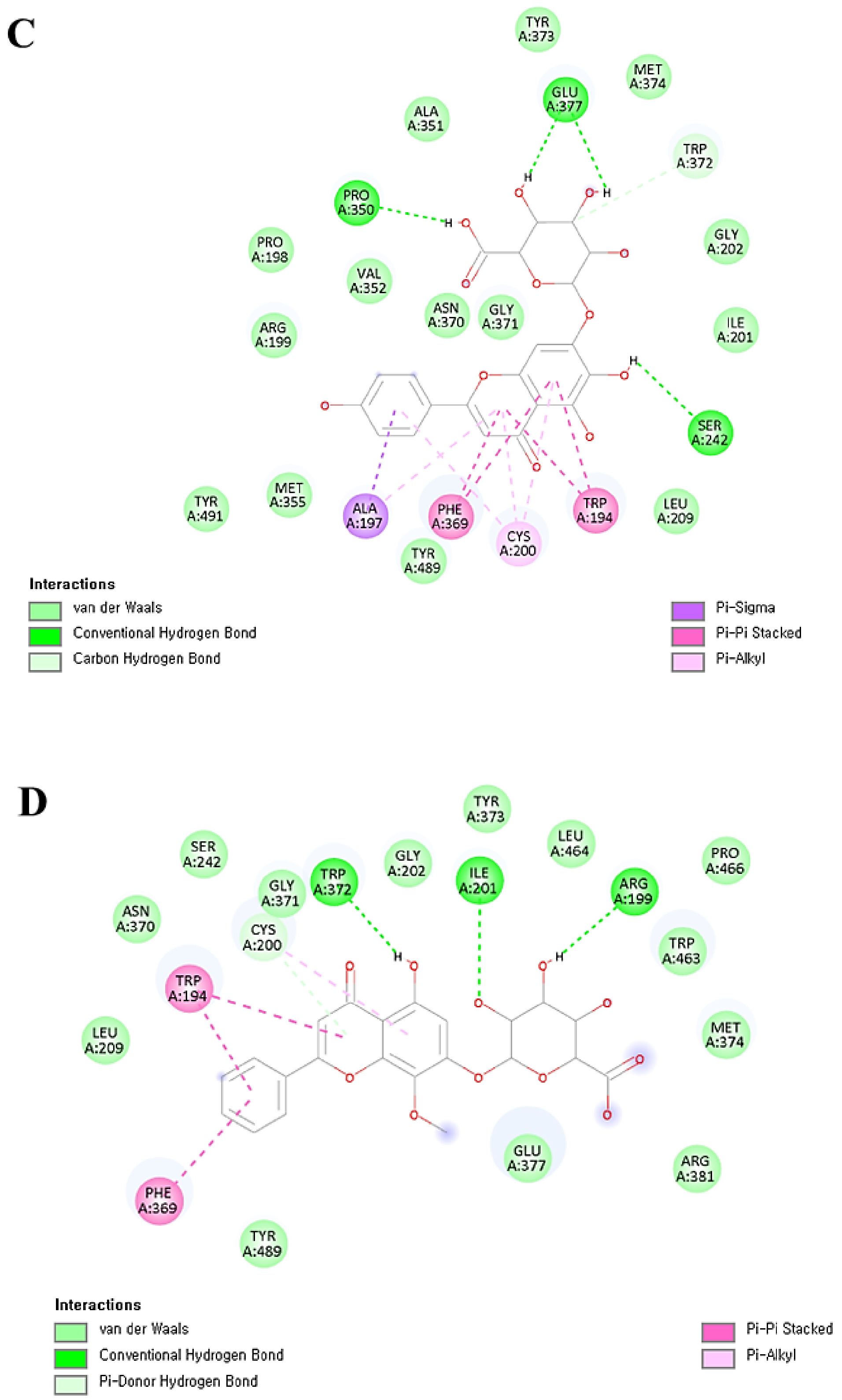

Figure S1. Two-dimensional (2D) representations showing the interaction types between NOS2 and four compounds of Huangqin decoction (HQD): baicalin (A), benzoylpaeoniflorin (B), scutellarin (C), and wogonoside (D).

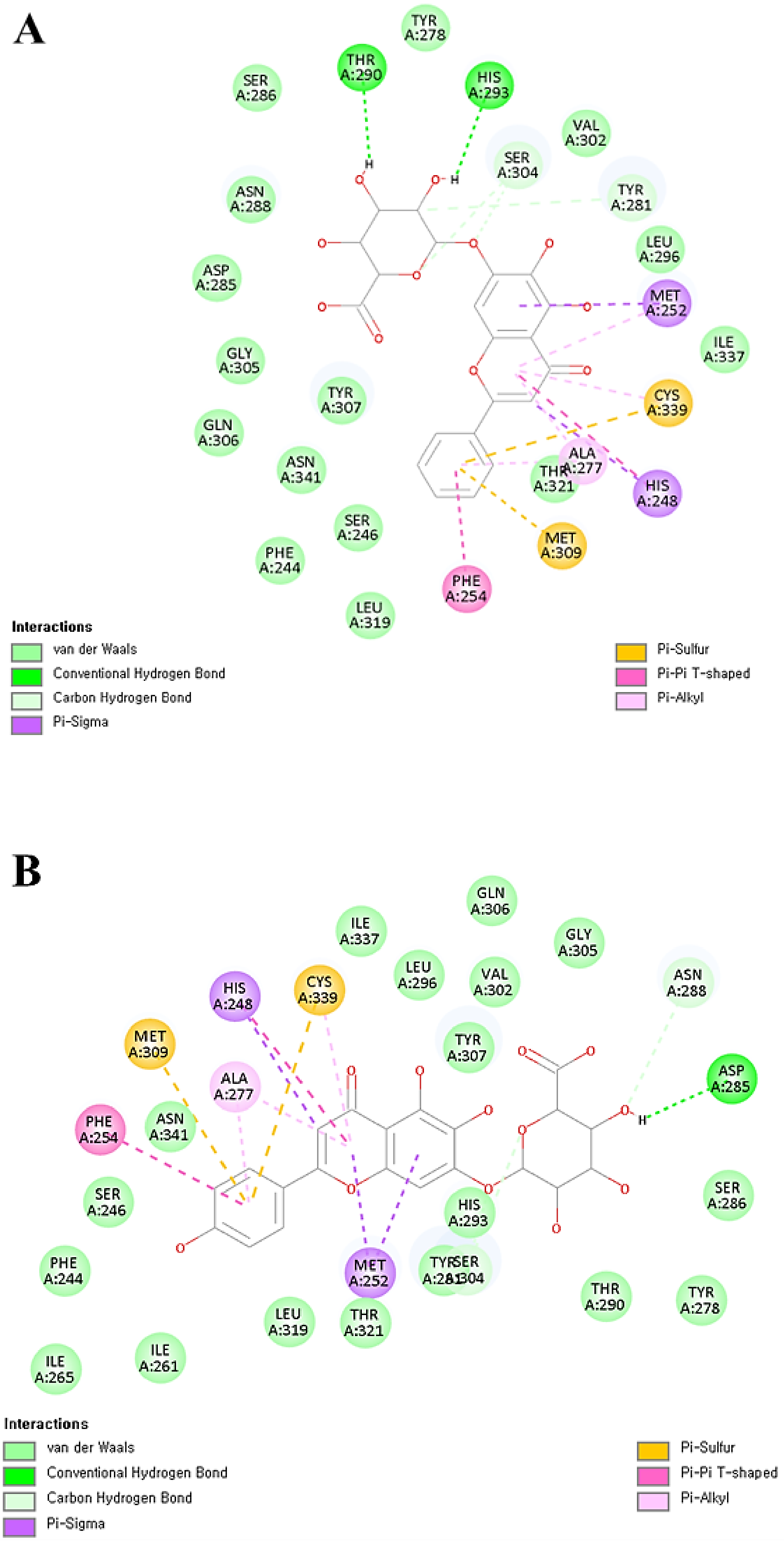
A
TYR A:278
THR A:290
HIS A:293
SER A:286
VAL A:302
SER A:304
ASN A:288
TYR A:281
LEU A:296
ASP A:285
MET A:252
ILE A:337
GLY A:305
TYR A:307
CYS A:339
GLN A:306
ASN A:341
ALA A:277
THR A:321
HIS A:248
SER A:246
MET A:309
PHE A:244
PHE A:254
LEU A:319
Interactions
van der Waals
Conventional Hydrogen Bond
Carbon Hydrogen Bond
Pi-Sigma
Pi-Sulfur
Pi-Pi T-shaped
Pi-Alkyl
B
GLN A:306
ILE A:337
GLY A:305
LEU A:296
VAL A:302
ASN A:288
HIS A:248
CYS A:339
MET A:309
TYR A:307
ASP A:285
ALA A:277
PHE A:254
ASN A:341
SER A:246
HIS A:293
SER A:286
PHE A:244
MET A:252
SER A:304
THR A:290
TYR A:278
LEU A:319
THR A:321
ILE A:265
ILE A:261
Interactions
van der Waals
Conventional Hydrogen Bond
Carbon Hydrogen Bond
Pi-Sigma
Pi-Sulfur
Pi-Pi T-shaped
Pi-Alkyl

C

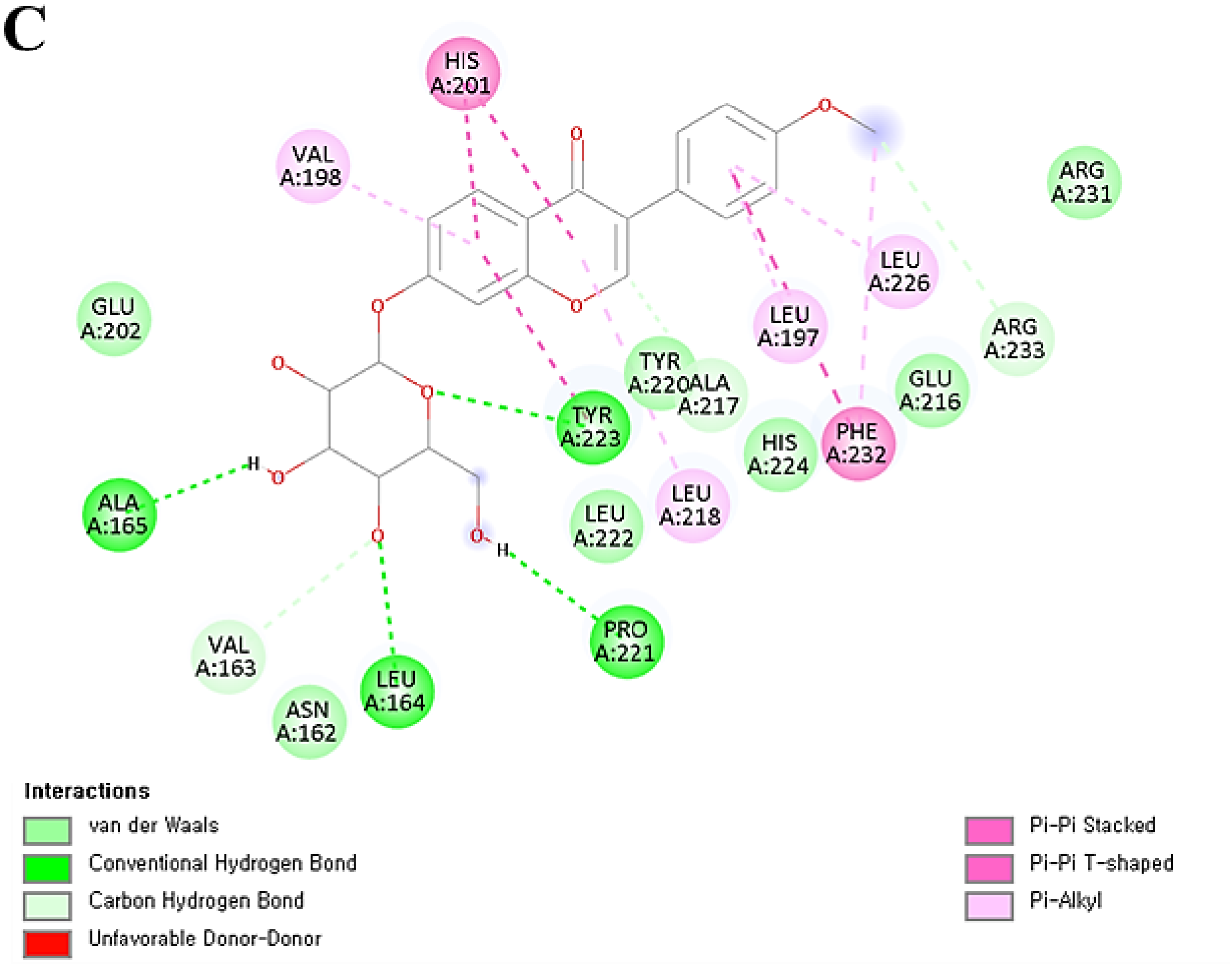
HIS A:201
VAL A:198
ARG A:231
LEU A:226
LEU A:197
ARG A:233
GLU A:202
TYR A:220
ALA A:217
GLU A:216
TYR A:223
HIS A:224
PHE A:232
ALA A:165
LEU A:218
LEU A:222
PRO A:221
VAL A:163
LEU A:164
ASN A:162
Interactions
van der Waals
Conventional Hydrogen Bond
Carbon Hydrogen Bond
Unfavorable Donor-Donor
Pi-Pi Stacked
Pi-Pi T-shaped
Pi-Alkyl

D

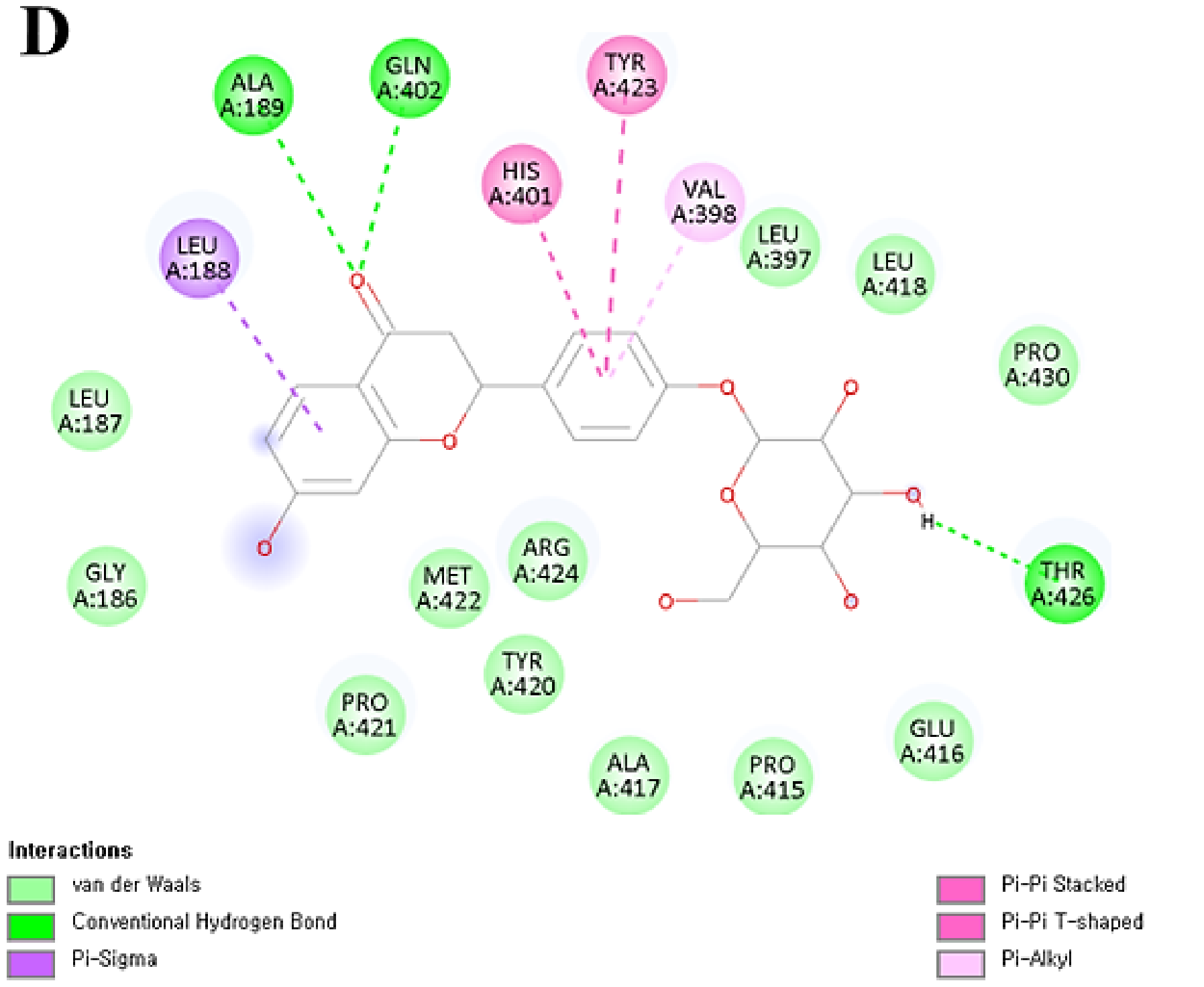
ALA A:189
GLN A:402
TYR A:423
HIS A:401
VAL A:398
LEU A:397
LEU A:418
LEU A:188
PRO A:430
LEU A:187
GLY A:186
MET A:422
ARG A:424
THR A:426
TYR A:420
PRO A:421
GLU A:416
ALA A:417
PRO A:415
Interactions
van der Waals
Conventional Hydrogen Bond
Pi-Sigma
Pi-Pi Stacked
Pi-Pi T-shaped
Pi-Alkyl

E

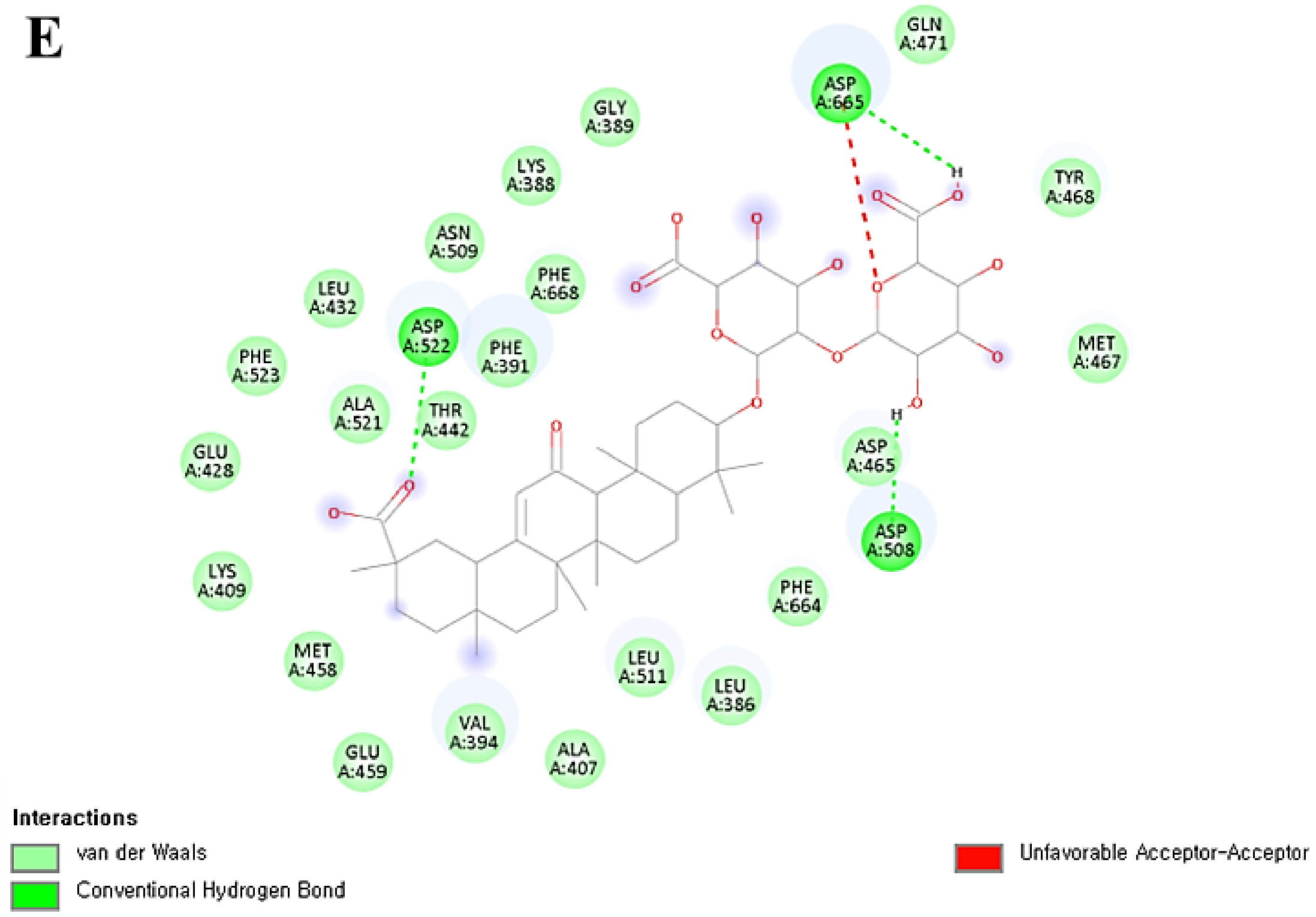

Figure S2. Two-dimensional (2D) representations of representative ligand–protein docking complexes exhibiting favorable docking scores: baicalin–AHR (A), scutellarin–AHR (B), ononin–MMP3 (C), liquiritin–MMP9 (D), and glycyrrhizic acid–PRKCQ (E).
